# Increased Nitric Oxide Bioavailability and Decreased Sympathetic Modulation Are Involved in Vascular Adjustments Induced by Low-Intensity Resistance Training

**DOI:** 10.3389/fphys.2016.00265

**Published:** 2016-06-28

**Authors:** Fabrício N. Macedo, Thassio R. R. Mesquita, Vitor U. Melo, Marcelo M. Mota, Tharciano L. T. B. Silva, Michael N. Santana, Larissa R. Oliveira, Robervan V. Santos, Rodrigo Miguel dos Santos, Sandra Lauton-Santos, Marcio R. V. Santos, Andre S. Barreto, Valter J. Santana-Filho

**Affiliations:** ^1^Laboratory of Cardiovascular Pharmacology, Department of Physiology, Federal University of SergipeSao Cristovao, Brazil; ^2^Laboratory of Cardiovascular Biology and Oxidative Stress, Department of Physiology, Federal University of SergipeSao Cristovao, Brazil; ^3^Department of Healthy Education, Estacio Faculty of SergipeAracaju, Brazil

**Keywords:** autonomic nervous system, resistance training, endothelium-dependent relaxing factors, nitric oxide, eNOS enzyme, nNOS enzyme

## Abstract

Resistance training is one of the most common kind of exercise used nowadays. Long-term high-intensity resistance training are associated with deleterious effects on vascular adjustments. On the other hand, is unclear whether low-intensity resistance training (LI-RT) is able to induce systemic changes in vascular tone. Thus, we aimed to evaluate the effects of chronic LI-RT on endothelial nitric oxide (NO) bioavailability of mesenteric artery and cardiovascular autonomic modulation in healthy rats. Wistar animals were divided into two groups: exercised (Ex) and sedentary (SED) rats submitted to the resistance (40% of 1RM) or fictitious training for 8 weeks, respectively. After LI-RT, hemodynamic measurements and cardiovascular autonomic modulation by spectral analysis were evaluated. Vascular reactivity, NO production and protein expression of endothelial and neuronal nitric oxide synthase isoforms (eNOS and nNOS, respectively) were evaluated in mesenteric artery. In addition, cardiac superoxide anion production and ventricle morphological changes were also assessed. *In vivo* measurements revealed a reduction in mean arterial pressure and heart rate after 8 weeks of LI-RT. *In vitro* studies showed an increased acetylcholine (ACh)-induced vasorelaxation and greater NOS dependence in Ex than SED rats. Hence, decreased phenylephrine-induced vasoconstriction was found in Ex rats. Accordingly, LI-RT increased the NO bioavailability under basal and ACh stimulation conditions, associated with upregulation of eNOS and nNOS protein expression in mesenteric artery. Regarding autonomic control, LI-RT increased spontaneous baroreflex sensitivity, which was associated to reduction in both, cardiac and vascular sympathetic modulation. No changes in cardiac superoxide anion or left ventricle morphometric parameters after LI-RT were observed. In summary, these results suggest that RT promotes beneficial vascular adjustments favoring augmented endothelial NO bioavailability and reduction of sympathetic vascular modulation, without evidence of cardiac overload.

## Introduction

Although well known the development of muscular strength, endurance, and mass, resistance exercise it is also related with reduction of cardiovascular disease risk. Currently, the inclusion of resistance exercise has been recommended by the American Heart Association (Pollock et al., 2000) and the American College of Sports Medicine (American College of Sports Medicine, 2009) as an important non-pharmacological strategy for the prevention and treatment of cardiovascular-related disorders as, hypertension and heart failure. Indeed, it is known that aerobic and resistance training (RT) can promote substantial benefits in physical fitness and health-related factors. Moreover, several studies reported that long-term of RT is able to reduce systolic and diastolic arterial pressure in cardiovascular diseased patients (Kelley and Kelley, 2000; Cornelissen and Smart, 2013).

Although coordinated, autonomic modulation and local signaling pathways, mostly controlled by smooth muscle and endothelial cells, play a pivotal role on the control of vascular tone and consequently, in the regulation of blood pressure (Garland et al., 1995; Thomas, 2011). Despite individual actions, it has been extensively demonstrated the relationship between abnormal endothelial dysfunction and higher incidence of cardiovascular events in diseased patients and in healthy population (Heitzer et al., 2001; Matsuzawa et al., 2013). Indeed, endothelial layer is composed by highly specialized cells which are able to release crucial modulators of vascular tone such as, endothelium-derived hyperpolarizing factors, prostacyclin and nitric oxide (NO) (Ignarro, 1989; Garland et al., 1995). It has been shown that exercise training can improve endothelial function in vascular beds, mainly due to increased shear stress stimulus (Niebauer and Cooke, 1996; Harris et al., 2010). Enhanced endothelium-dependent vasorelaxation has been described after aerobic training due to increased NO bioavailability, reduction in the sensitivity of contractile agonists and vascular sympathetic modulation (Jansakul and Hirunpan, 1999; Higashi and Yoshizumi, 2004). However, the lack of studies dissecting the potential contribution of autonomic modulation and local intracellular mechanisms involved in the decrease of blood pressure induced by RT, encouraged the present study.

Besides of exercise modality, the effects of RT on vascular function seem to be dependent of exercise intensity. High-intensity RT has been associated with deleterious responses on the vascular function and structural remodeling (Cortez-Cooper et al., 2005). On the other hand, recent evidences have been reported that low-intensity RT is able to improve vascular endothelial function and peripheral blood circulation in healthy young and elderly population (Shimizu et al., 2016) and to reduce arterial stiffness (Okamoto et al., 2011, 2014). However, whether low-intensity RT induces a protective or maladaptive effect on vascular function is not yet well understood. Therefore, the aim of this study was to evaluate the participation of endothelial nitric oxide and autonomic modulation in the vascular tone adjustments, and consequently, blood pressure control induced by low-intensity resistance training in healthy rats.

## Materials and methods

### Animals

All experimental procedures were performed in accordance with the Guide for the Care and Use of Laboratory Animals published by National Institutes of Health (NIH, 8th edition) and approved by the Ethics Committee on Animal Research of the Federal University of Sergipe (#01/2013). Twenty-eight male Wistar rats (250–270 g, 12- to 14-weeks old) were obtained from the Animal Care Facility of the Federal University of Sergipe and housed under standard conditions (food and water *ad libitum*, 12:12 h light/dark cycle and ~22°C). The animals were assigned into two experimental groups: sedentary (SED, *n* = 14) and resistance trained (Ex, *n* = 14).

### Resistance training protocol

SED and Ex animals underwent a 1 week familiarization period (5 days, 5 min per day in rest position) in a customized squat apparatus for RT, as developed by Tamaki et al. (1992). Electrical stimulation (20 V, 0.3 s duration, at 3 s intervals) was applied on the tail of the rat through a surface electrode. After familiarization period, both groups were subjected to a one maximal repetition test (1RM) which consists to determine the maximum weight lifted by the rat in the exercise apparatus. The 1RM test was repeated every 2 weeks in attempt to maintain the desired intensity. Ex group was subjected to a RT protocol which consists in 3 sets of 10 repetitions with intensity defined at 40% of the maximum load established in the 1RM test, the animals were exercised three times per week (alternated days) for 8 weeks. SED group was subjected to a fictitious exercise consisting in a similar procedures and electrical stimulation as Ex group, however, without physical effort.

### *In vivo* measurements

The animals were anesthetized with a mixture of ketamine/xylasine (80 mg/kg and 10 mg/kg, respectively, i.p) and polyethylene catheter was implanted (PE-10/PE-50, Intramedic, Becton Dickinson and Company, Sparks, MD, USA) into the femoral artery. The catheter was tunneled and exteriorized in the posterior cervical region and the animals were allowed to recovery for 24 h. Afterwards, the catheter was connected to a pressure transducer (FE221, Bridge Amp, ADInstruments, Bella Vista, NSW, Australia) coupled to a pre-amplifier (Powerlab 8/35, AdInstruments). Values of mean arterial pressure (MAP), systolic arterial pressure (SAP), diastolic arterial pressure (DAP) and heart rate (HR) were obtained and assessed after 8 weeks of experimental procedures.

### Cardiovascular autonomic modulation

The baroreflex sensitivity (BRS) was measured in the time domain by the sequence method (Bertinieri et al., 1985). Series beat-to-beat were analyzed by software CardioSeries v2.4 (http://sites.google.com/site/cardioseries). Sequences of at least 4 heart beats with increased SAP followed by PI lengthening or subsequent decrease of SAP with PI shortening with correlation greater than 0.85 were identified as baroreflex sequence. The slope of the linear regression between SAP and PI was considered as a measure of BRS.

Cardiac autonomic balance was evaluated by frequency domain. The PI and SAP variability analysis were performed using the same software previously described. Series beat-to-beat were obtained by pulsatile arterial pressure and converted into points every 100 ms using cubic spline interpolation (10 Hz). The interpolated series were divided into half-overlapping sequential sets of 512 data points (51.2 s). Before calculation of the spectral power density, the segments were visually inspected and the non-stationary data were not taken into consideration. The spectrum was calculated from the Fast Fourier Transformation (FFT) algorithm direct and Hanning window was used to attenuate side effects. The spectrum is composed of bands of low frequency (LF; 0.2–0.75 Hz) and high frequency (HF; 0.75–3 Hz), the results were showed in normalized units, by calculating the percentage of the LF and HF variability with respect to the total power after subtracting the power of the very low frequency (VLF) component (frequencies < 0.20 Hz), namely Low Frequency/High Frequency (LF/HF) ratio.

The LF/HF ratio from pulse interval represents sympathovagal balance. LF and HF components mean cardiac sympathetic and parasympathetic activity. LF from systolic arterial pressure (LFsys) represents sympathetic vascular modulation (Task Force of the European Society of Cardiology and the North American Society of Pacing and Electrophysiology, 1996).

### Vascular reactivity

Animals were euthanized by an over-dose of thiopental (100 mg/kg) and the superior mesenteric artery was removed, cleaned from connective and fat tissues and sectioned into rings (1–2 mm). Rings were suspended by fine stainless hooks connected to a force transducer (Letica, Model TRI210; Barcelona, Spain) with cotton threads in organ baths containing Tyrode's solution (Composition in mM: NaCl 158.3, KCl 4.0, CaCl_2_ 2.0, NaHCO_3_ 10.0, C_6_H_12_O_6_ 5.6, MgCl_2_1.05, and NaH_2_PO_4_ 0.42). The solution was continually gassed with carbogen (95% O_2_ and 5% CO_2_) and maintained at 37°C under a resting tension of 0.75 g for 60 min (stabilization period). After 1 h of stabilization, endothelium-intact mesenteric rings were pre-contracted with phenylephrine (Phe, 1 μM) and cumulative concentration–response curves to vasodilator agonist, acetylcholine (ACh, 10^−9^–10^−4^ M), were performed in the absence or presence of a NOS inhibitor, N^ω^-nitro-L-arginine methyl ester (L-NAME, 100 μM). Phe-induced vasoconstriction (10^−6^ M) was also assessed in the absence or presence of L-NAME. Relaxing responses were plotted as percentage of the contraction induced by Phe. Vasoconstriction induced by Phe was expressed as maximal tension developed (grams).

### Measurements of NO production

NO production in the mesenteric artery was determined using a fluorescent cell-permeable dye, 4,-amino-5 methylamino-2′,7′-diaminofluorescein diacetate (DAF-FM; Molecular Probes), as previously described (Mota et al., 2015). Freshly isolated mesenteric arteries were loaded with 10 μM of the dye for 40 min at 37°C in Tyrode solution, 20 min after the onset of the probing, some rings were stimulated with 1 μM of ACh and then washed for 40 min. Mesenteric segments were frozen in medium for cryosectioning and cut into 20 μm thick sections. Images were recorded using a fluorescence microscope (Ci-E, Nikon, Japan) under identical settings. The fluorescent intensity was measured using ImageJ software (NIH). A minimum of 10 regions were randomly selected in the endothelial and smooth muscle layers from each mesenteric section. It worthwhile note that smooth muscle exhibits an autofluorescence, therefore, in order to avoid misleading fluorescence measurements, analyses of images were carefully performed selecting the region of interest between the smooth muscle fibers. Fluorescence microscopy images were analyzed according to the intensity of the fluorescence per area and the data are expressed as arbitrary unit (a.u.).

### Measurements of reactive oxygen species generation

Hearts were quickly removed and frozen in medium for cryosectioning. Frozen tissue blocks were transversely cut into 30 μm thick sections and slides transferred to a recording chamber. Slides were pre-incubated with warmed Tyrode solution for 15 min and then, the sections were loaded with 5 μM of dihydroethidium, a fluorescent cell-permeable dye (DHE; Molecular Probes) for 30 min (Erickson et al., 2008). DHE staining with light fixation was adapted from a published method (Owusu-Ansah et al., 2008). Images were recorded using a fluorescence microscope (Ci-E, Nikon, Japan) and the fluorescence intensity was measured using ImageJ software (NIH).

### Western blot

Western blot was performed as described previously (Mota et al., 2015). Mesenteric artery was homogenized in ice-cold lysis buffer containing (in mM): 150 NaCl, 50 Tris-HCl, 5 EDTA.2 Na and 1 MgCl2, pH 8.0; 1% Triton X-100, 1% NP-40, 1% sodium deoxycholate, 0.1% sodium dodecyl sulfate enriched with a protease inhibitor cocktail (Sigma FAST, Sigma, St. Louis, MO). Homogenates were cleared by centrifugation at 13,000 × g for 15 min at 4°C and protein content was quantified by Lowry assay. Samples were denaturated in Laemmli's buffer and equal amount of protein (30 μg/lane) was separated on 10% SDS-polyacrylamide gel electrophoresis and then, transferred onto nitrocellulose membrane (2 h at 120 V, Merck-Millipore, Billerica, MA). Membranes were blocked for 2 h in Tris-buffered saline-Tween 20 containing 5% non-fat dry milk at room temperature before incubation with rabbit polyclonal anti-eNOS, anti-nNOS and goat polyclonal anti-GAPDH antibodies (1:1000, Santa Cruz Biotechnology, Santa Cruz, CA) overnight at 4°C. After washing and incubation for 2 h at room temperature with anti-rabbit or anti-goat IgG-HRP antibody (1:10,000, Sigma-Aldrich, St. Louis, Missouri, USA), immunodetection was performed using enhanced chemiluminescence (Luminata strong™—Western HRP substrate, Merck-Millipore, Billerica, MA). Digitalized images were analyzed by densitometry using ImageJ software (NIH).

### Cardiac morphometry

Heart hypertrophy was evaluated by morphometry. After anesthesia, heart beat was stopped in diastole using 10% KCl (i.v.). Hearts were embedded in medium for cryosectioning and frozen at −80°C. Transversal sections (5 μm) were cut starting from apex to base of the heart and stained with hematoxylin-eosin for cardiac morphometry. Tissue sections (3–6 for each animal) were examined with (Ci-E, Nikon, Japan) microscope and analyzed with ImageJ software.

### Statistical analysis

All data are expressed as mean ± S.E.M. Significant differences between groups were determined using Two-way ANOVA followed by Bonferroni's *post-hoc* test to compare the concentration-response curves obtained in mesenteric rings. One-way ANOVA followed by Bonferroni's *post-hoc* test was used to compare the Phe-elicited vasoconstriction and NO production. Student's paired *t*-test was used to compare 1RM and bodyweight intragroup. Student's unpaired *t*-test was used for all other analysis. All statistical comparisons were made using GraphPad Prism 5.1 (GraphPad Software Inc., San Diego, CA, USA) and values of *P* < 0.05 were considered to be statistically significant.

## Results

### Exercise training efficacy

As shown in the Table 1, all groups showed a body weight gain during the study period; however, 8 weeks of low-intensity RT promoted a smaller body weight gain compared to the SED group. Moreover, Ex group showed a significant enhancement on physical fitness assessed by increased 1RM/Body Weight (BW) ratio.

**Table 1:** **Bodyweight (BW), one maximal repetition test values (1RM) and 1RM/BW ratio before and after 8 weeks of low-intensity resistance training in healthy rats**.

		**SED (*n* = 8)**	**Ex (*n* = 8)**
Bodyweight (g)	Initial	262 ± 4.6	262 ± 4.5
	Final	314 ± 7.1	295 ± 4.4^*^
1RM (g)	Initial	1165 ± 61	1305 ± 69
	Final	1800 ± 75	2030 ± 68^*^
1RM/Bodyweight	Initial	4.44 ± 0.29	4.90 ± 0.36
	Final	5.73 ± 0.29	6.88 ± 0.45^*^^†^

SED, Sedentary group; Ex, Exercised group; 1RM, One-Repetition Maximum Test. Values expressed as mean ± S.E.M. To evaluate differences between groups and intragroup (final vs. initial), it were used unpaired (^*^) and paired (†) T-Test, respectively.

*, † *p < 0.05*.

### Hemodynamic parameters, morphometry of the left ventricle and oxidative stress biomarker

**Figure 1:**
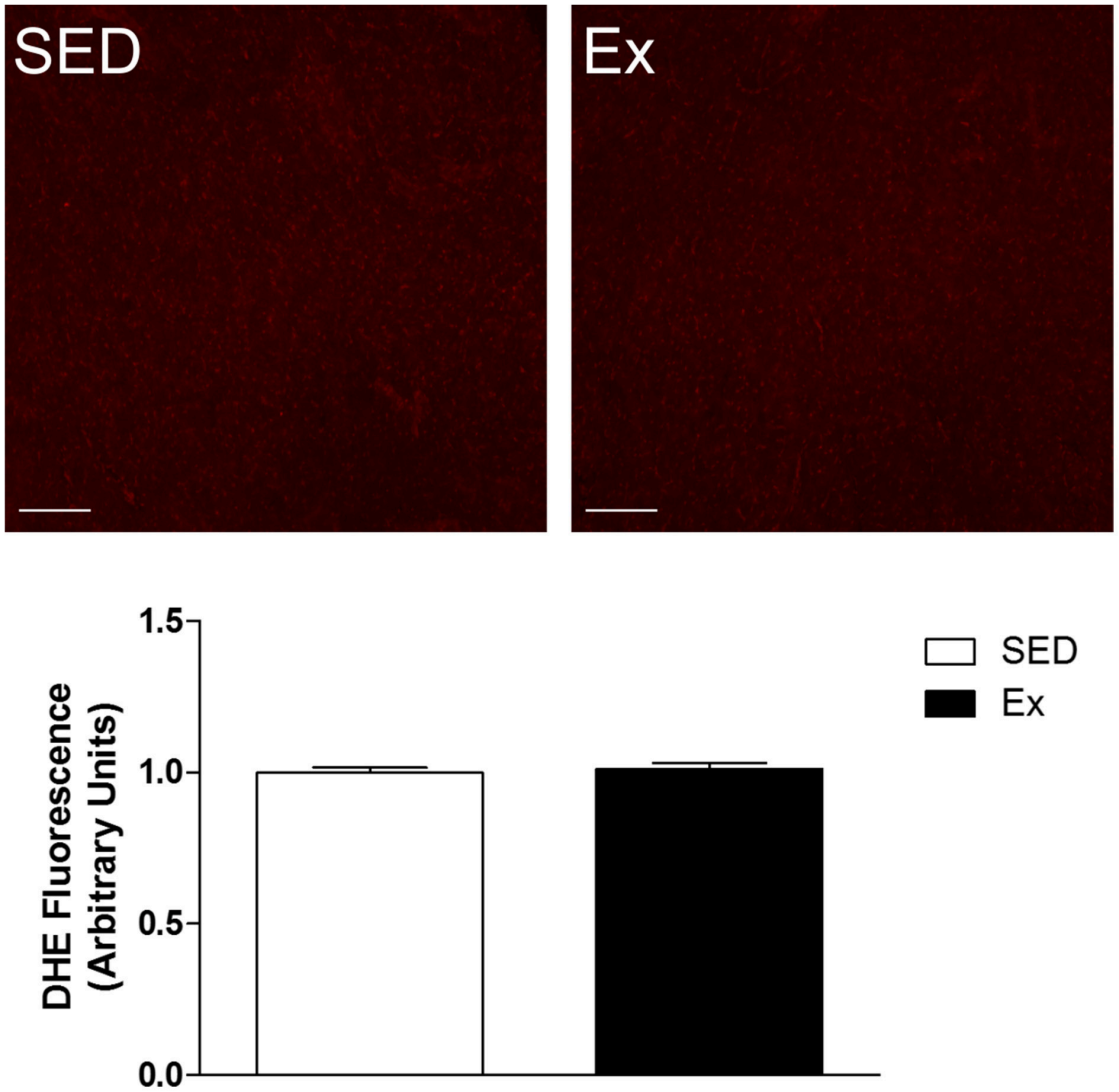
**Effects of low-intensity resistance training on left ventricle superoxide production of healthy rats**. Top panel, representative images of DHE fluorescence, a ROS indicator and Bottom panel, quantification of DHE fluorescence in left ventricle myocardium slides of sedentary (SED) and exercise-trained (Ex) rats. Scale bar = 20 μm. Values are expressed as mean ± S.E.M for 3–5 left ventricle slides analyzed from 3 rats in each group. For data analysis, Student's unpaired *t*-test was used.

The hemodynamic parameters, morphometric analysis of left ventricular myocardium and reactive oxygen species generation measurements were evaluated at the end of the experimental protocol. As shown in the Table 2, Ex group showed a significant reduction in mean (MAP), systolic (SAP) and diastolic (DAP) arterial pressures, associated with decreased heart rate (HR), when compared with SED group. It is worth mentioning that the hemodynamic measurements were performed in conscious rats. On the other hand, RT neither induced cardiac hypertrophy nor superoxide generation changes compared with SED group, as shown in Table 2 and Figure 1, respectively.

**Table 2:** **Hemodynamic parameters and morphology of the left ventricle after 8 weeks of low-intensity resistance training in healthy rats**.

		**SED (*n* = 8)**	**Ex (*n* = 8)**
MAP (mmHg)		117.04 ± 2.68	105.5 ± 4.28^*^
SAP (mmHg)		136.71 ± 4.92	122.5 ± 4.24^*^
DAP (mmHg)		107.71 ± 2.95	97.0 ± 3.42^*^
HR (bpm)		395 ± 7.10	344 ± 13.25^**^
LVED (mm^2^)		620 ± 31	560 ± 23
LVPWT (cm)		0.30 ± 0.02	0.37 ± 0.02
LVID (mm^2^)		8.13 ± 1.01	7.37 ± 0.97

*SED, Sedentary group; Ex, Exercised group; MAP, Mean Arterial Pressure; SAP, Systolic Arterial Pressure; DAP, Diastolic Arterial Pressure; HR Heart Rate; LVED, left ventricle external diameter; LVPWT, left ventricle posterior wall thickness; LVID, left ventricle internal diameter. Values expressed as mean ± S.E.M. To evaluate differences between groups, it was used unpaired T-test*.

* p < 0.05 and

** *p < 0.01 when compared SED vs. Ex*.

### Vascular reactivity

**Figure 2:**
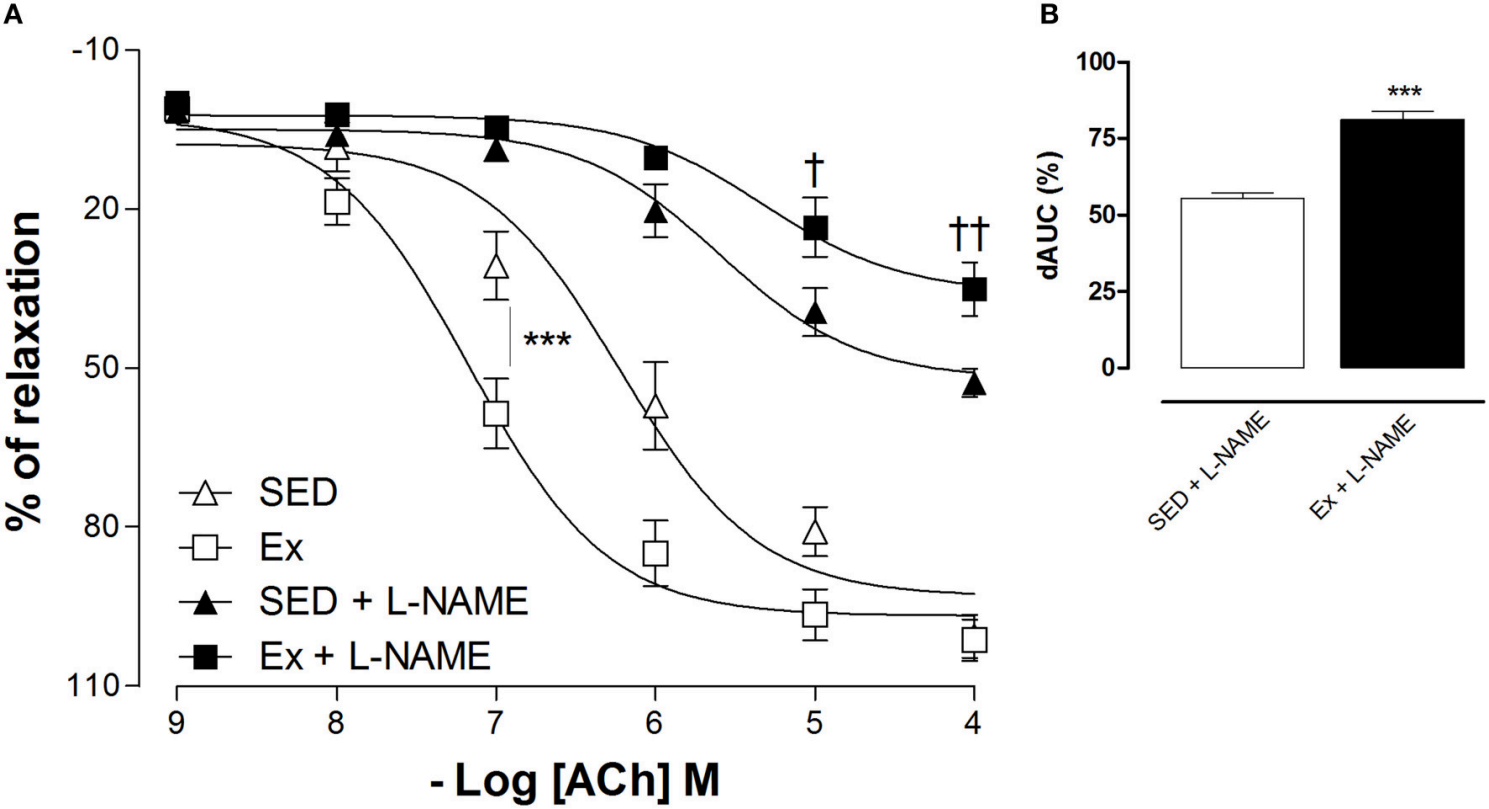
**Effects of low-intensity resistance training on endothelium-dependent relaxation of healthy rats**. Cumulative concentration-response curves for ACh in mesenteric artery of sedentary (SED) and exercise-trained (Ex) rats in the absence or presence of L-NAME (100 μM, **A**). The difference in the area under the concentration–response curve (dAUC, **B**) for ACh-induced relaxation is expressed as a percentage of decrease in the AUC before and after pre-incubation with L-NAME. Values are expressed as mean ±S.E.M. for 6 experiments in each group. For concentration-response curve, statistical differences were determined by two-way ANOVA followed by Bonferroni's test and for dAUC, Student's unpaired *t*-test was used. ^***^p < 0.001, Ex vs. SED; ^†^*p* < 0.05 and ^††^*p* < 0.01, Ex+L-NAME vs. SED+L-NAME.

**Figure 3:**
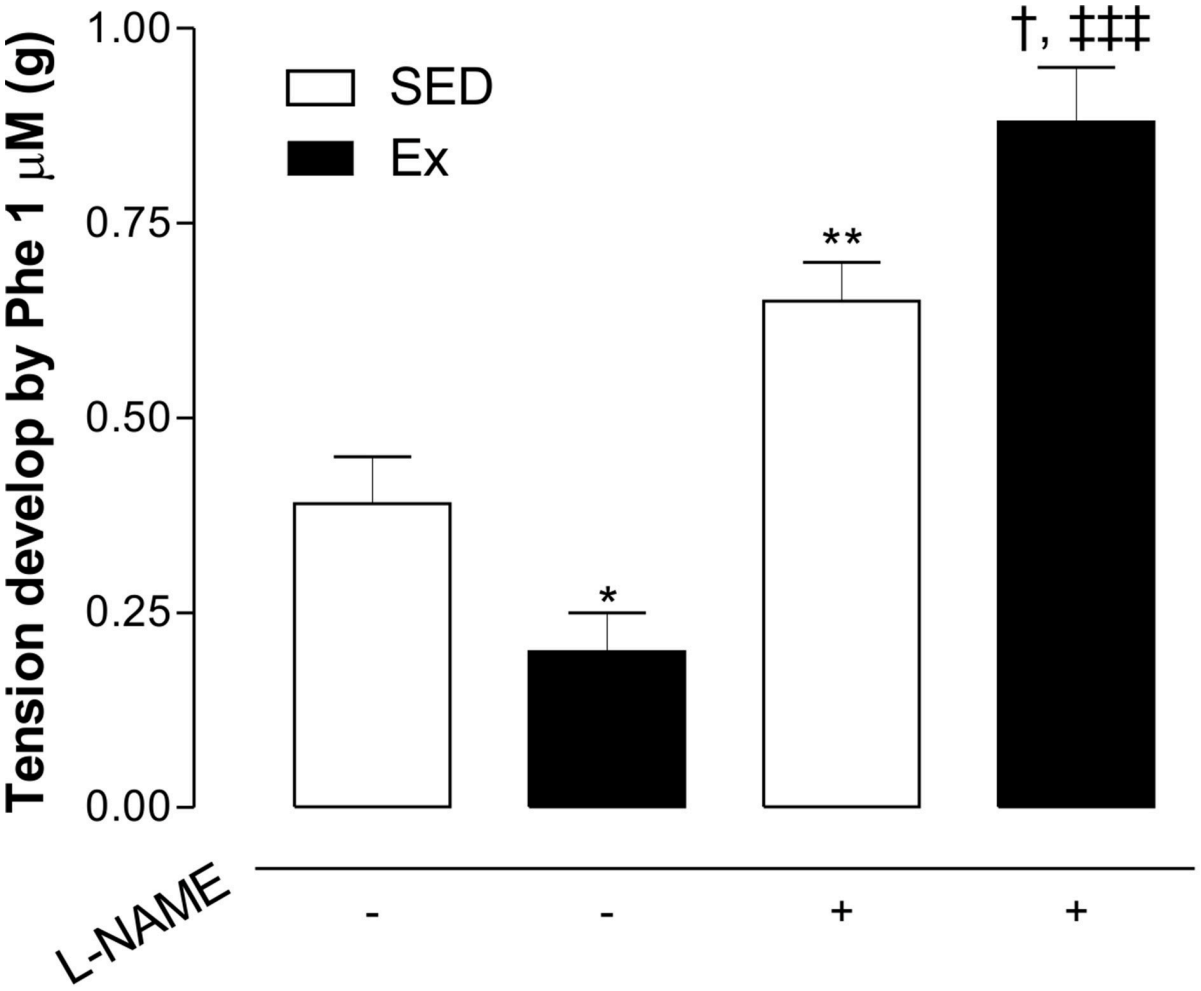
**Effects of low-intensity resistance training on Phe-induced vasoconstriction of healthy rats**. The developed tension elicited by Phe (1 μM) was evaluated in mesenteric artery of sedentary (SED) and exercise-trained (Ex) rats in the absence or presence of L-NAME (100 μM). Values are expressed as mean ± S.E.M. for 6 experiments in each group. To evaluate difference between groups, it was used one-way ANOVA followed by Bonferroni post-test. ^*^p < 0.05 and ^**^p < 0.01, vs. SED without L-NAME pre-incubation (−); ^†††^*p* < 0.001, vs. EX without L-NAME pre-incubation; †*p* < 0.05, vs. SED with L-NAME pre-incubation.

Based on hemodynamic findings, we assessed whether RT affects the endothelium-dependent relaxation of mesenteric artery. As expected, ACh induced a concentration-dependent relaxation in all groups, however, 8 weeks of low-intensity RT induced a significant leftward shift of ACh-induced relaxation in Ex group compared to SED group (Figure 2A; pD_2_ values: Ex group, 7.1 ± 0.1 and SED group, 6.2 ± 0.1). Maximal relaxant response (Rmax) to ACh was similar between Ex and SED rats (Figure 2A). To evaluate the involvement of NO on the enhanced ACh-induced vasorelaxation promoted by RT, inhibition of both constitutive NOS, eNOS and nNOS, markedly reduced the Rmax in SED and Ex group. However, the inhibition was significantly higher in Ex group than SED group. To further explore these results, the difference of the area under the curve (dAUC) demonstrates a greater dependence of NO for ACh-induced vasorelaxation in Ex (81.17 ± 2.7%) than SED group (55.4 ± 5.2%; Figure 2B). Furthermore, as shown in the Figure 3, the vasoconstriction induced by Phe was decreased in Ex group (0.20 ± 0.05 g) compared with SED group (0.39 ± 0.06 g). In accordance with ACh vasorelaxation response, L-NAME significantly potentiates the vasoconstriction response of Phe in Ex group (0.88 ± 0.07 g) and SED (0.65 ± 0.05 g), however, the developed tension was higher in Ex group than SED group.

### NO bioavailability and constitutive NOS expression

**Figure 4:**
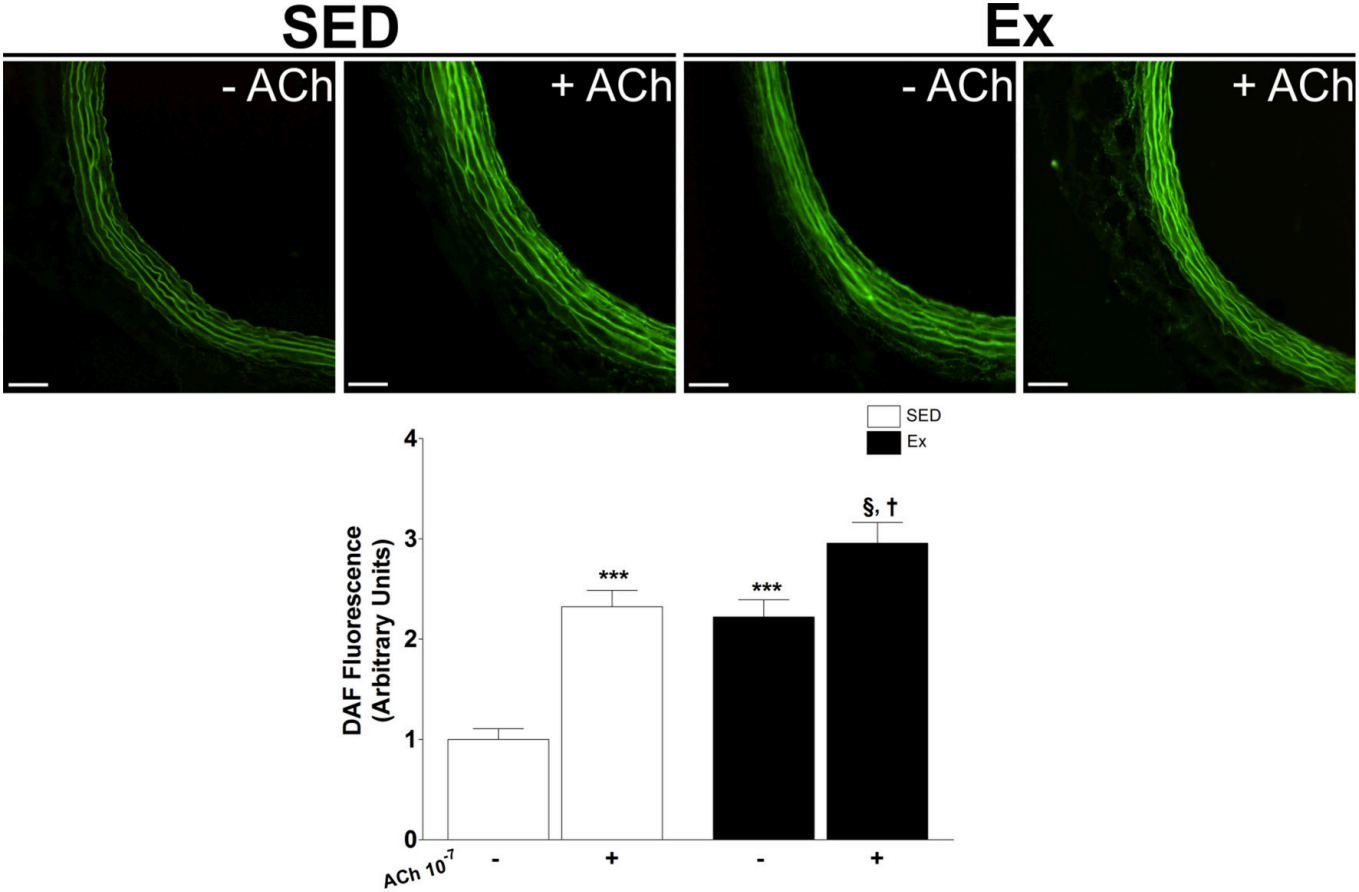
**Effects of low-intensity resistance training on basal and ACh-stimulation nitric oxide (NO) production in superior mesenteric artery**. Top panel, representative images of DAF fluorescence, a NO indicator and Bottom panel, quantification of DAF fluorescence in mesenteric artery of sedentary (SED) and exercise-trained (Ex) rats. Scale bar = 20 μm. Values are expressed as mean ± S.E.M. for 30–40 mesenteric rings analyzed from 3 animals in each group. To evaluate difference between groups, it was used one-way ANOVA followed by Bonferroni post-test. ^***^p < 0.001, vs. SED under basal condition (−); ^§^*p* < 0.05, vs. Ex under basal condition (−); ^†^*p* < 0.05 vs. SED under ACh stimulation (+).

**Figure 5:**
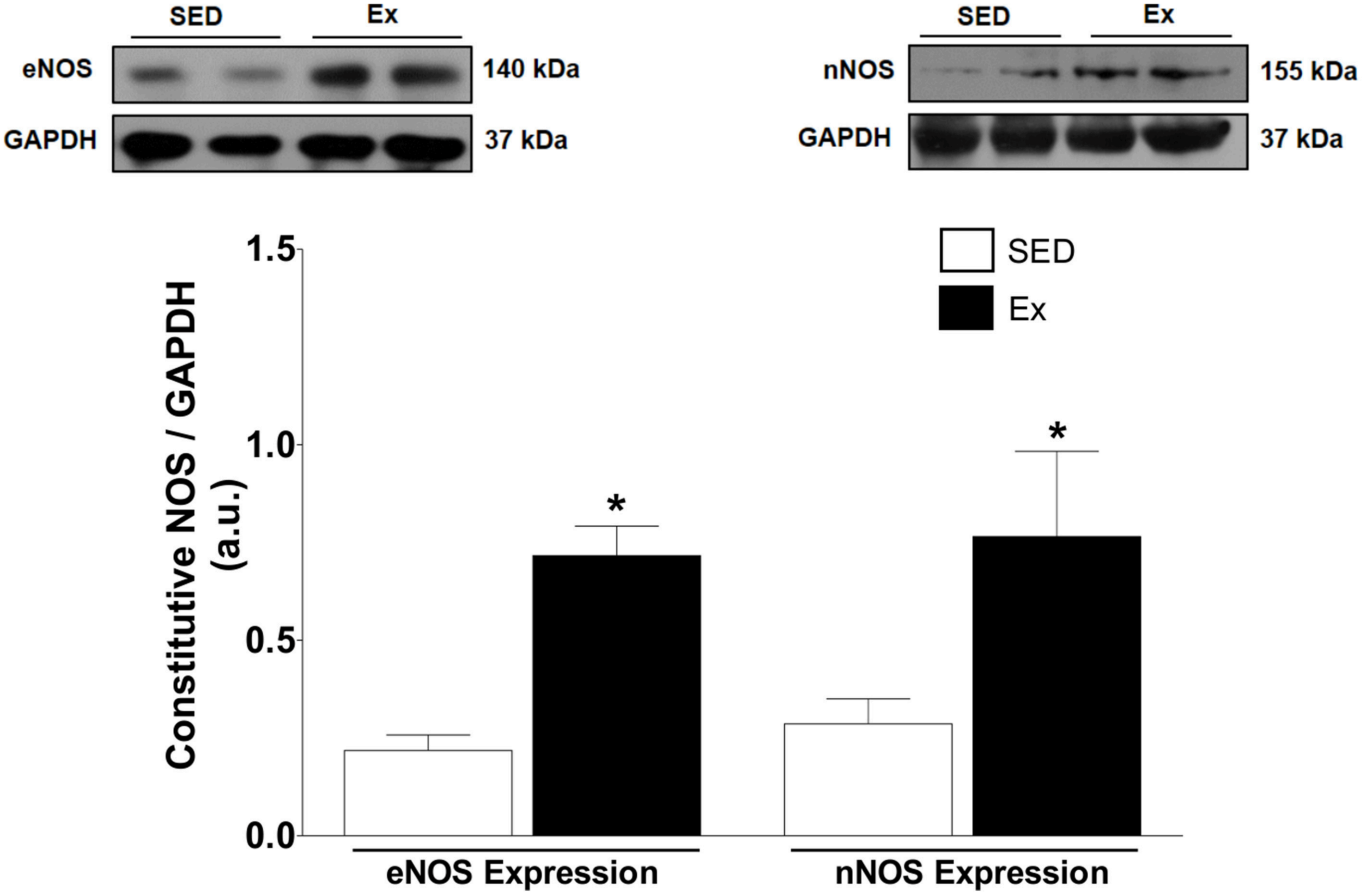
**Effects of low-intensity resistance training on constitutive NOS expression of healthy rats**. Top panel, representative images of the western blot and Bottom panel, quantitative analysis of total eNOS and nNOS protein level in mesenteric artery of sedentary (SED) and exercise-trained (Ex) rats. Values are expressed as mean ± S.E.M for 5 different samples in each group. For data analysis, Student's unpaired *t*-test was used. ^*^p < 0.05 Ex vs. SED.

Based on our *in vitro* findings, we next assessed whether RT affects NO synthesis and protein expression of eNOS and nNOS in mesenteric artery. Interesting, NO production under basal condition was 1.2-fold higher in Ex group than in SED group (Figure 4). It is well known that NO production is the result of eNOS and nNOS activities in endothelial cells. Therefore, we indirectly tested the activities of NOS in endothelium-intact mesenteric artery, monitoring the end-product released by active NOS when stimulated for 20 min with ACh (1 μM). It is worth mentioning that the adopted concentration of ACh was determined based on pD_2_ values found in our *in vitro* vascular reactivity data. Figure 4 shows that ACh stimulation elicited a marked increase on NO production in Ex e SED rats. However, NO production was significantly higher in Ex group than SED group when stimulated with ACh. Consequently, we next investigated whether the expression of both constitutive NOS enzymes were altered after 8 weeks of low-intensity RT in mesenteric artery. In accordance with our findings, western blot analysis revealed increased protein expression levels of both, eNOS (~2.4-fold) and nNOS (~1.7-fold), in Ex when compared to SED rats (Figure 5).

### Neural control of blood pressure

**Figure 6:**
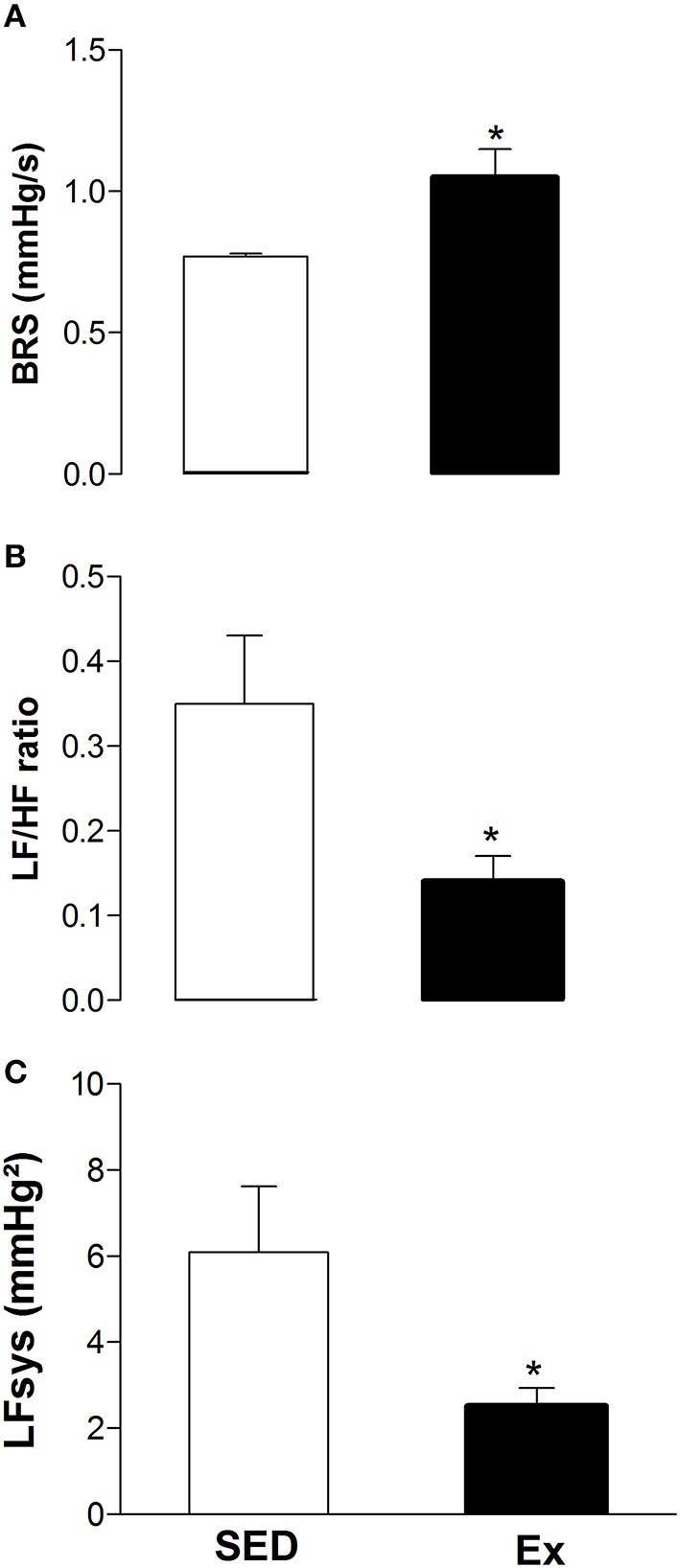
**Effects of low-intensity resistance training on neural control of blood pressure of healthy rats. (A)** Spontaneous arterial baroreflex sensitivity (BRS); **(B)** power spectral analysis of heart rate (LF/HF ratio) and **(C)** arterial pressure (LFsys) variability. Sedentary (SED) and Exercise-trained (Ex) rats. Values are expressed as mean ± S.E.M. for 6 experiments in each group. For data analysis, Student's unpaired *t*-test was used. ^*^p < 0.05 Ex vs. SED.

It is well known that the autonomic nervous system plays a crucial role in blood pressure control. Therefore, we next evaluated whether RT affects arterial baroreflex sensitivity and cardiovascular variability after 8 weeks of low-intensity RT. As shown in the Figure 6A, the spontaneous baroreflex sensitivity (BRS) was significantly increased after RT when compared to SED rats. However, RT was able to reduce the power spectral analysis of heart rate (LF/HF ratio; Figure 6B) and arterial pressure variability (LFsys; Figure 6C) when compared to SED rats.

## Discussion

In this study, the effect of 8 weeks of low-intensity resistance training (LI-RT) on the hemodynamic parameters, cardiac oxidative stress, left ventricular myocardium remodeling, vascular adjustments and autonomic control were evaluated in normotensive rats. The major findings are: (1) Decrease in both blood pressure and heart rate with no cardiac overload; (2) Improvement of endothelium-dependent vascular function, which is correlated with increased endothelial NO bioavailability and upregulation of eNOS and nNOS in mesenteric artery; (3) Reduction in vasoconstrictor responsiveness to phenylephrine; (4) Enhanced spontaneous baroreflex sensitivity accompanied by reduction in a cardiac and vascular sympathetic modulation.

The model of resistance training used in this work involves a caudal electrical stimulation to accomplish exercise movement (Tamaki et al., 1992). A recent study of our laboratory demonstrated that caudal electrical stimulation isolated is not able to induce changes in vascular response after a single bout of RT (Fontes et al., 2014). In addition, Barauna et al. (2005) showed that stressor parameters, such as plasma catecholamines or adrenal weight did not suffer any change by electrical stimulation isolated. Therefore, the cardiovascular results found in this work are attributed solely to LI-RT.

Muscle strength due a RT protocol are strongly associated with workload. It is well known that muscle hypertrophy and neuromuscular adaptation can enhance this physical valence. Gabriel et al. (2006) demonstrates that RT, independent of the intensity used, develops an important neuromuscular component, however, the size of this adaptation are intensity dependent. LI-RT protocol used in our study were able to increase the maximal weight lifted in Ex group. Even in low intensity, a period of RT was an effective method to increase muscular strength, however there was no difference in body weight between groups. Despite skeletal muscle adaptation, high and moderate intensities of resistance exercise are associated with acute and chronic deleterious cardiovascular effects such as, increase in the arterial stiffness, (Miyachi et al., 2004; Miyachi, 2013) cardiac hypertrophy (Barauna et al., 2005, 2007), arterial baroreflex and vascular sympathetic dysfunction (Niemelä et al., 2008; Collier et al., 2009). On the other hand, LI-RT has been demonstrated as an efficient and safer exercise to be applied to health and pathological populations in humans (Okamoto et al., 2011, 2014) or experimental model (Mostarda et al., 2014).

The LI-RT protocol here adopted consisted in low workload (40% of 1RM), moderated number of repetitions and short sets to minimize pressure overload. It has been shown that pressure overload induces a great production of reactive oxygen species inducing the oxidative stress, which ultimately result in cardiac hypertrophy (Takimoto and Kass, 2007; Brown and Griendling, 2015). In the present study, 8 weeks of LI-RT was not able to induce changes in the superoxide generation nor left ventricular remodeling. High levels of cardiac oxidation are implicated with pathogenesis and aggravation of several cardiometabolic diseases as hypertension, heart failure and diabetes (Lakshmi et al., 2009; Fiorentino et al., 2013). In fact, a number of studies indicated the cardioprotective actions of physical exercise due increased activity and expression of antioxidants enzymes in the heart (Chicco et al., 2006; Azizbeigi et al., 2014; Borges and Lessa, 2015). In contrast, it has been shown left ventricle hypertrophy as consequence of RT performed at high intensity (Huonker et al., 1996; Barauna et al., 2007). Taken together, the absence of oxidative stress and morphological changes in the left ventricular myocardium suggest that the practice of resistance training under low intensity does not develop cardiac risk factors.

It is well known that RT has important impact on hemodynamics parameters reducing the blood pressure in normotensive, hypertensive and diabetics individuals (Kelley and Kelley, 2000; Eves and Plotnikoff, 2006; Cornelissen et al., 2011; Moraes et al., 2012). In humans, recent evidences indicate that acute high intensity resistance exercise seems to be effective in lowering blood pressure in healthy young and hypertensive elderly population (Brito et al., 2014; Duncan et al., 2014). Similarly, Queiroz et al. (2015) showed that low intensity of resistance exercise intensities led to changes in blood pressure in healthy and hypertension population. However, as previously described, maladaptive changes are triggered by high intensity RT which could limit the efficacy–safety ratio of resistance exercise.

In the present study, we observed that 8 weeks of LI-RT reduced mean, systolic and diastolic blood pressure, as well as heart rate. In general, hemodynamic effects of RT have been associated with changes in local signaling pathways and neurohumoral mechanisms (Katz et al., 1997; Selig et al., 2004; Figueroa et al., 2008; Speretta et al., 2016). Accordingly with our hemodynamic findings and consistent with clinical human evidence (Okamoto et al., 2014), we demonstrated that LI-RT enhances the vasorelaxation response in superior mesenteric artery of trained rats. However, knowing that the vascular tone is resultant of the balance between competing vasodilator and vasoconstrictor factors, we found a decrease in contractile responses to phenylephrine. Based on our data, the mechanism that underlies the increase in responsiveness to ACh, it is suggestive to propose an increased endothelial NO bioavailability in mesenteric artery of trained rats.

As expected, the non-selective NOS inhibitor markedly decreased the endothelium-dependent relaxation induced by ACh and potentiated the Phe-induced vasoconstriction. Surprisingly, the magnitude of these changes was higher in RT animals. As previously demonstrated by our group, acute high intensity RT was also able to activate MAPK/ET-1 pathway, a vasoconstrictor mechanism; however, taking into account the difference between the intensity and acute effect, we suggest that this mechanism might be involved in the potentiated vascular contractile response found in RT rats under NOS inhibition. Moreover, these findings support the notion of the higher dependence of NO bioavailability in mesenteric artery of trained rats.

Confirming our hypothesis, basal production of NO was higher in mesenteric arteries of RT rats. It has been extensively described the pivotal role of eNOS-derived NO from endothelial cells for vascular relaxation (Förstermann and Sessa, 2012). However, although less studied, nNOS-derived relaxing factors such as, NO and H_2_O_2_, also contribute to endothelium-dependent relaxation (Capettini et al., 2010). Here, we indirectly test the whole constitutive NOS activities in intact-endothelium mesenteric arteries monitoring the additional synthesis of NO when stimulated with ACh. Hence, a marked increase in ACh-induce NO synthesis were found; however, LI-RT potentiated the production of NO. Therefore, to unequivocally demonstrate the involvement of constitutive NOS in the vascular effects induced by LI-RT, protein expression analysis demonstrated an upregulation of eNOS and nNOS in mesenteric artery of RT rats. Previous study has demonstrated the participation of eNOS and nNOS in the control of vascular tone (Nangle et al., 2004), however, the role of nNOS in the endothelial dysfunction and hypertension is extremely limited. Thus, this study provides important insights related to the understanding of vascular adjustments induced by RT and, although not investigated, we suggest that nNOS/NO axis might be involved in the therapeutic actions of resistance exercise.

Importantly, the vascular bed here studied regulates about 20% of total blood flow in the body and changes in its vascular perfusion can represents significant alterations in total vascular peripheral resistance (Blanco-Rivero et al., 2013). Therefore during exercise, mesenteric arteries, suffer a large decrease in the blood flow, described as an intensity-dependent phenomenon known as reactive hyperemia (Joyner and Casey, 2015). Although recommended by several healthcare guidelines, intracellular mechanisms related with resistance exercise response is still largely unexplored. On the other hand, several studies have proposed eNOS/NO, Pi3K/Akt and AMPK signaling as the main intracellular pathways involved the protective cardiovascular actions of the aerobic exercise (McMullen et al., 2007; Wang et al., 2010; Cacicedo et al., 2011). Taken together, the present study added a valuable information about the systemic adaptation induced by LI-RT describing the importance of the expression and functionality of eNOS and nNOS in the vascular adjustments.

Studies have shown that NO can influence autonomic control function by several mechanisms (Patel et al., 1996; Lin et al., 2007; Schultz, 2009). Two important mechanisms postulated are: 1. NO can increase arterial baroreflex sensitivity and 2. NO in nucleus tractus solitarii causes sympathoinhibitory effect, blocking the rostral ventrolateral medulla (Schultz, 2009). In addition, it is known that arterial baroreflex and vascular sympathetic activity are associated with increased NO bioavailability in vascular beds (Kolo et al., 2004; Gamboa et al., 2007; Conti et al., 2013). Although we utilized indirect measurements of autonomic control, our results showed a higher spontaneuous baroreflex sensitivity and lower cardiac and vascular sympathetic modulation in LI-RT rats. Interestingly, these mechanisms may contribute to a high NO bioavailability, inhibiting the metabolization and release of noradrenergics neurotransmitters on vascular sympathetic nerve endings (Greenberg et al., 1990; Daveu et al., 1997; Kolo et al., 2004). Gmitrov (2015), observed other important effect of NO-baroreflex axis, demonstrated by the baroreflex-mediated increament in sensitivity to NO. Taken all together, we belive that baroreflex-NO axis works in a state of positive feedback promoting a decrease in heart and vascular sympathetic modulation and increasing cardiovascular safety.

In summary, our results suggest that LI-RT promotes a decrease in the blood pressure associated with enhanced Ach-induced vasorelaxation response in rats mesenteric artery. More importantly, our results clearly show that these changes are followed by an upregulation of eNOS and nNOS leading to elevated NO bioavailability in vascular tissue. Besides of that, spontaneuous baroreflex sensitivity improvement followed by reduction of cardiovascular sympathetic modulation also contribute to lower values of basal blood pressure and bradycardia. Such evidence confirm that LI-RT is able to promote systemic cardiovascular adjustments with no evidences of cardiovascular overload, being considered safer to be applied as a non-pharmacological strategy to health maintenance.

## Author contributions

FM, TM, VM, MM, TS, MS, LO, RS, and Rd performed experiments, data analyses and drafted the manuscript. SL, MRVS, AB, and VS designed the study and contributed to the manuscript. All authors approved the final version of the manuscript.

## Funding

This study was supported by Conselho Nacional de Desenvolvimento Científico e Tecnológico (CNPq, Brazil)—Coordenação de Aperfeiçoamento de Pessoal de Nível Superior (CAPES, Brazil) and Fundação de Apoio à Equipe e à Inovação Tecnológica do Estado de Sergipe (FAPITEC/SE—Brazil).

### Conflict of interest statement

The authors declare that the research was conducted in the absence of any commercial or financial relationships that could be construed as a potential conflict of interest. The reviewer JC and handling Editor declared their shared affiliation, and the handling Editor states that the process nevertheless met the standards of a fair and objective review
